# Separating Glioma Hyperintensities From White Matter by Diffusion-Weighted Imaging With Spherical Tensor Encoding

**DOI:** 10.3389/fnins.2022.842242

**Published:** 2022-04-21

**Authors:** Jan Brabec, Faris Durmo, Filip Szczepankiewicz, Patrik Brynolfsson, Björn Lampinen, Anna Rydelius, Linda Knutsson, Carl-Fredrik Westin, Pia C. Sundgren, Markus Nilsson

**Affiliations:** ^1^Medical Radiation Physics, Lund University, Lund, Sweden; ^2^Diagnostic Radiology, Lund University, Lund, Sweden; ^3^Department of Radiology, Brigham and Women’s Hospital, Harvard Medical School, Boston, MA, United States; ^4^Division of Medical Radiation Physics, Department of Translational Medicine, Lund University, Lund, Sweden; ^5^Department of Neurology, Lund University, Lund, Sweden; ^6^Russell H. Morgan Department of Radiology and Radiological Science, Johns Hopkins University School of Medicine, Baltimore, MD, United States; ^7^F.M. Kirby Research Center for Functional Brain Imaging, Kennedy Krieger Institute, Baltimore, MD, United States; ^8^Lund University Bioimaging Center, Lund University, Lund, Sweden; ^9^Department of Imaging and Physiology, Skåne University Hospital, Lund University, Lund, Sweden

**Keywords:** diffusion MRI, glioma, white matter (WM), hyperintensity, spherical encoding, isotropic encoding, conspicuity, detection

## Abstract

**Background:**

Tumor-related hyperintensities in high *b*-value diffusion-weighted imaging (DWI) are radiologically important in the workup of gliomas. However, the white matter may also appear as hyperintense, which may conflate interpretation.

**Purpose:**

To investigate whether DWI with spherical b-tensor encoding (STE) can be used to suppress white matter and enhance the conspicuity of glioma hyperintensities unrelated to white matter.

**Materials and Methods:**

Twenty-five patients with a glioma tumor and at least one pathology-related hyperintensity on DWI underwent conventional MRI at 3 T. The DWI was performed both with linear and spherical tensor encoding (LTE-DWI and STE-DWI). The LTE-DWI here refers to the DWI obtained with conventional diffusion encoding and averaged across diffusion-encoding directions. Retrospectively, the differences in contrast between LTE-DWI and STE-DWI, obtained at a *b*-value of 2,000 s/mm^2^, were evaluated by comparing hyperintensities and contralateral normal-appearing white matter (NAWM) both visually and quantitatively in terms of the signal intensity ratio (SIR) and contrast-to-noise ratio efficiency (CNR_eff_).

**Results:**

The spherical tensor encoding DWI was more effective than LTE-DWI at suppressing signals from white matter and improved conspicuity of pathology-related hyperintensities. The median SIR improved in all cases and on average by 28%. The median (interquartile range) SIR was 1.9 (1.6 – 2.1) for STE and 1.4 (1.3 – 1.7) for LTE, with a significant difference of 0.4 (0.3 –0.5) (*p* < 10^–4^, paired *U*-test). In 40% of the patients, the SIR was above 2 for STE-DWI, but with LTE-DWI, the SIR was below 2 for all patients. The CNR_eff_ of STE-DWI was significantly higher than of LTE-DWI: 2.5 (2 – 3.5) vs. 2.3 (1.7 – 3.1), with a significant difference of 0.4 (−0.1 –0.6) (*p* < 10^–3^, paired *U*-test). The STE improved CNR_eff_ in 70% of the cases. We illustrate the benefits of STE-DWI in three patients, where STE-DWI may facilitate an improved radiological description of tumor-related hyperintensity, including one case that could have been missed out if only LTE-DWI was inspected.

**Conclusion:**

The contrast mechanism of high *b*-value STE-DWI results in a stronger suppression of white matter than conventional LTE-DWI, and may, therefore, be more sensitive and specific for assessment of glioma tumors and DWI-hyperintensities.

## Introduction

Diffusion-weighted imaging (DWI) is central to the diagnostic workup for patients with glioma (Barajas et al., 2016; Kolakshyapati et al., 2017; Zeng et al., 2018; White et al., 2019). A sign of poor prognosis is the presence of tumor hyperintensities on the DWIs, which are often referred to as regions of reduced diffusivity. The proposed underlying pathophysiological mechanism is a link between high tumor cellularity and reduced diffusivity (Chen et al., 2013; Laviolette et al., 2014). With stronger diffusion weightings (*b* = 2,000–3,000 s/mm^2^), the hyperintense regions are more conspicuous (Seo et al., 2008; Zeng et al., 2018), more useful for discriminating high- and low-grade gliomas (Seo et al., 2008), and better for prediction of the overall survival for patients with glioblastomas (Zeng et al., 2018). In a recent study, we observed that glioma conspicuity might be further improved by computing diffusion metrics based on so-called spherical tensor encoding (STE) (Nilsson et al., 2020), which at high *b*-values, yields an image contrast that is different from that obtained with conventional DWI (Szczepankiewicz et al., 2015).

Conventional DWI encodes for diffusion, along a single direction, at the time of the application of a pair of pulsed gradients (Stejskal and Tanner, 1965). Radiologic evaluation is performed by visual inspection of the DWI averaged over diffusion directions or by quantitative evaluation of the apparent diffusion coefficient (ADC) or its directional average—the mean diffusivity (MD). The MD is negatively correlated with tumor cellularity (Chenevert et al., 2000; Surov et al., 2017). The quantification of MD typically relies on DWI, with a moderate diffusion-weighting (*b* ≈ 1,000 s/mm^2^). When stronger diffusion-weightings are used (*b* ≈ 2,000–3,000 s/mm^2^), we may also estimate the mean kurtosis (MK) (Jensen et al., 2005), which is useful in glioma imaging because it enables their typing and grading (Tietze et al., 2015; Delgado et al., 2017).

Conventional DWI suffers from an inherent limitation, as it attenuates the MR signal in proportion to diffusion, but only along a single diffusion-encoding direction at a time. When the signal attenuation is low, we can only infer that diffusivity was reduced in that direction. However, distinctly different types of tissues can give rise to reduced diffusion. For example, a tightly packed dense tissue with high cellularity will exhibit low ADC, but so will the anisotropic structures when diffusion-encoding is applied along their short axis. If such anisotropic structures are not coherently aligned, we will find low ADC in many directions (Szczepankiewicz et al., 2016). Thus, the regions with high diffusion-weighted signal intensity in the directional average may reflect high anisotropy, as well as dense tissue with high cellularity. This problem arises because the conventional diffusion-weighted signal is sensitive to both isotropic diffusivity and diffusion anisotropy.

Tensor-valued diffusion encoding can be used to separate isotropic diffusivity and diffusion anisotropy by introducing a new measurement dimension—the b-tensor shape—that can be varied to obtain more information on the microstructure (Mitra, 1995; Mori and Van Zijl, 1995; Wong et al., 1995; Shemesh and Cohen, 2011; Eriksson et al., 2013; Lawrenz and Finsterbusch, 2013; Lasič et al., 2014; Szczepankiewicz et al., 2015; Henriques et al., 2021). Using the b-tensor terminology (Westin et al., 2016), a conventional DWI employs linear b-tensor encoding (LTE) as it encodes for diffusion along a single direction at a time. These images acquired with LTE are often orientationally averaged during post-processing (here referred to as LTE-DWI). By contrast, spherical b-tensor encoding (STE) sensitizes the signal to diffusion in all directions at a time (Lasič et al., 2014; Szczepankiewicz et al., 2016). The STE-DWI is, thus, already averaged across orientations during the acquisition. More importantly, however, is that STE attenuates anisotropic structures in proportion to their isotropic diffusivity so that a high signal is obtained only in regions with a low isotropic diffusivity, which is expected in tightly packed dense tissue with high cellularity. This means that STE-DWI is weighted for average isotropic diffusivity only but conventional DWI (LTE-DWI) for the combination of isotropic diffusivity and diffusion anisotropy. This effect is prominent at higher *b*-values. Conventional DWI entangles these two features even when it appears to be isotropic diffusion on the voxel scale. That is because this could be due to diffusion in isotropic structures or incoherent ordered anisotropic structures. Note that STE-DWI cannot be obtained from conventional LTE-DWI such as by the trace of the diffusion tensor imaging (DTI). The STE-DWI has previously been used to highlight the difference in cell density between the cortices of the cerebrum and cerebellum (Lundell et al., 2019; Tax et al., 2020; Vis et al., 2021) or for fast MD mapping (Mori and Van Zijl, 1995; Wong et al., 1995; Ianuş and Shemesh, 2018; Lasič et al., 2020; Szczepankiewicz et al., 2021a), but has not yet been systematically explored in gliomas.

The main hypothesis of this study is that the glioma tumors have higher conspicuity to the white matter at high *b*-value DWI obtained with STE (referred to as STE-DWI) than with high *b*-value DWI obtained with LTE and averaged across several directions (referred to as LTE-DWI). This is founded on the principle that gliomas exhibit reduced diffusion and low diffusion anisotropy because they are composed of densely-packed and approximately spherical cells, whereas the white matter is composed of elongated axons, which exhibit intermediate diffusivity and high anisotropy, as previously described (Beaulieu, 2002; Szczepankiewicz et al., 2015). Due to the orientation dispersion in white matter, we may never encode in a direction parallel with all axons in a voxel (Jeurissen et al., 2013). This causes LTE to yield less attenuation in white matter and reduces the contrast between white matter regions and regions with reduced isotropic diffusion. With STE, however, the MR signal of each part of the voxel is attenuated in proportion to its isotropic diffusivity. Accordingly, the white matter will appear darker (hypointense) with STE-DWI than LTE-DWI. Relative to the white matter, regions with reduced isotropic diffusivity will appear brighter (hyperintense) with STE-DWI (Szczepankiewicz et al., 2016). To test if gliomas are more conspicuous with STE-DWI than with LTE-DWI, we evaluated the contrast differences between LTE-DWI and STE-DWI both visually and quantitatively in a cohort of patients with glioma.

## Materials and Methods

### Patients

This retrospective study included 25 patients with a brain tumor, who underwent an MRI examination, as part of a larger research study of patients who were radiologically diagnosed with a brain tumor. The project was approved by the local ethics committee-the Regional Ethical Review Board in Lund (2016/531, 2017/866 and 2018/993), and written informed consent was obtained from each volunteer according to recommendations of the declaration of Helsinki. The inclusion criteria, for this study, were above 18 years of age, histologically confirmed glioma tumor verified after surgery or biopsy or by known histopathological diagnosis after previous surgical resection for the tumor (in cases of recurrent tumor), and completed an MRI examination with at least one pathology-related hyperintensity on both LTE-DWI and STE-DWI at *b*-value 2,000 s/mm^2^. Furthermore, for comparison, we also included a patient without a brain tumor or other MRI abnormality except for a vascular malformation (telangiectasia) in the right temporal lobe outside of the regions that were evaluated (referred to as patient C1). We also included a patient that met all the inclusion criteria but did not have a hyperintensity present on the DWI scans (referred to as patient C2).

### MRI Acquisition

Imaging was performed on a MAGNETOM Prisma 3 T system (Siemens Healthcare, Erlangen, Germany) with a 20-channel head-neck coil. The DWI was performed, before the administration of a gadolinium-containing contrast agent, using a prototype diffusion-weighted spin-echo sequence (Szczepankiewicz et al., 2019), with a repetition time (TR) of 5,000 ms, echo time (TE) 84 ms, echo spacing 0.7 ms, transversal slices, read-out bandwidth 1,612 Hz/pixel, phase direction anterior-posterior, pre-scan normalization off, iPAT 2 (GRAPPA), partial Fourier 6/8 (number of readout lines was 37), fat saturation “strong” (including gradient reversal fat suppression), resolution 2.3 mm × 2.3 mm × 2.3 mm, and field-of-view (FOV) of 230 mm × 230 mm × 85 mm. The data were acquired with two types of b-tensors (LTE and STE) and *b*-values of 100, 700, 1,400, and 2,000 s/mm^2^. These *b*-values were acquired in 3, 3, 6, and 6 directions with LTE. For STE, the same *b*-values were instead repeated at 6, 6, 11, and 16 times, respectively. The diffusion encoding was performed using asymmetric gradient waveforms that were numerically optimized to minimize TE (Sjölund et al., 2015), “max norm” gradient bounds, heat dissipation factor of 0.5, and a slew rate limit of 50 T/m/s. The optimization software is available at https://github.com/jsjol/NOW. The STE waveforms were not rotated (Szczepankiewicz et al., 2021b), but their amplitude was modulated to yield different *b*-values. The total scan time of the tensor-valued dMRI sequence (including both LTE and STE) was 5 min. The data subset that included only the images at *b* = 2,000 s/mm^2^ had an acquisition time of 30 s for LTE and 80 s for STE. Moreover, the T_1_-weighted 3D magnetization prepared rapid gradient echo (MPRAGE) before and after contrast administration, and T_2_-weighted luid-attenuated inversion recovery (FLAIR) images were acquired at a resolution of 1 mm × 1 mm × 1 mm.

### Data Post-processing

The diffusion-weighted images were corrected for eddy currents and motion with ElastiX (Klein et al., 2009) using extrapolated target volumes (Nilsson et al., 2015). The MD and S_0_ maps were manually corrected for failed fitting of some individual voxels that were predominately present in the cerebrospinal fluid (CSF), and their values were replaced by values from neighboring voxels. To make signal levels comparable across brain regions, we performed bias-field correction on the LTE-DWI and STE-DWI volumes using the FMRIB’s automated segmentation tool (FAST) method from the FMRIB software library (Zhang et al., 2001). All images were co-registered using ElastiX to the post-gadolinium T_1_-weighted images by a rigid-body transformation that included up-sampling of the diffusion-weighted images to T_1_-weighted image space.

### Qualitative Analysis of Hyperintensity-White Matter Relation

We investigated if hyperintensities have higher conspicuity to white matter with STE-DWI than LTE-DWI. To test this qualitatively, we compared the appearances of the LTE-DWI and STE-DWI at *b* = 2,000 s/mm^2^ around hyperintensities unrelated to the white matter, as well as in normal white matter. We corroborated our findings with the co-registered T_1_-weighted images acquired before and after the administration of gadolinium, T_2_-FLAIR, MD maps, and DWI images acquired without diffusion encoding (*S*_0_). To assess in which anatomical locations the STE-DWI yields the highest attenuation in comparison with LTE-DWI, we visualized the signal difference between LTE-DWI and STE-DWI in the patient without MRI abnormalities in the regions evaluated (referred to as patient C1).

### Quantitative Analysis of Hyperintensity-White Matter Relation

For the quantitative analysis, we drew for each patient’s regions-of-interest (ROIs) overall hyperintensities, and contralateral normal-appearing white matter (NAWM). The hyperintensity ROIs were delineated on co-registered and up-sampled images obtained with LTE-DWI only (here disregarding STE-DWI) and were defined as regions with the high signal at *b* = 2,000 s/mm^2^ over or in the near vicinity of contrast-enhancement regions on T_1_-weighted images, or hyperintense regions on T_2_-FLAIR images. For each patient, we also drew ROIs on the contralateral NAWM that encompassed both large and small white matter tracts to factor out sensitivity to different white matter regions. The ROIs were drawn by J.B. (physician) and reviewed by F.D. (physician). We extracted the signal values of LTE-DWI and STE-DWI from both the hyperintensity and NAWM ROIs and evaluated four quantities. In the first and second, we investigated the differences of the signal values between LTE-DWI and STE-DWI in the hyperintensity and NAWM ROIs. In the third, we calculated signal intensity ratios (SIR), defined as the ratio between the mean value of the signal within the ROIs of the hyperintensities and the NAWM according to:


(1) $$\text{SIR}\,\left(X\right)=\frac{\left\langle S_{\mathrm{hyperintensity}}\,\left(X\right)\right\rangle }{\left\langle S_{\mathrm{NAWM}}\,\left(X\right)\right\rangle }$$

where the brackets ⟨⋅⟩ represent the averaging of signal values within the ROI, and *X* refers to the modality (either LTE-DWI or STE-DWI). In the fourth, we calculated the contrast-to-noise ratio efficiency (CNR_eff_) between the hyperintensity and NAWM, defined as:


(2) $${\mathrm{CNR}}_{\mathrm{eff}}\,\left(X\right)=\frac{\left\langle S_{\mathrm{hyperintensity}}\,\left(X\right)\right\rangle -\left\langle S_{\mathrm{NAWM}}\,\left(X\right)\right\rangle }{\sigma_{N}},$$

where the standard deviation (SD) σ_*N*_ refers to the standard deviation of the noise per acquisition. Thus, it is independent of the number of averages, which enables a comparison of LTE-DWI or numbers these were acquired with different number of directions or averages, respectively. In practice, σ_*N*_ was estimated as the mean within the NAWM ROI of the standard deviation of all 16 repetitions of STE-DWI at *b* = 2,000 s/mm^2^. The estimation was based on the STE-DWI, and not LTE-DWI, because LTE-DWI was acquired from several directions and, thus, the signal varies due to both noise and directional information. By contrast, variations in signal intensity across the STE acquisitions was only due to noise as these were acquired with identical settings. Note that σ_*N*_ is not the standard deviation of the image noise from the conventional definition of the contrast-to-noise ratio (CNR). The conveninal CNR can be computed as $\mathrm{CNR}={\mathrm{CNR}}_{\text{eff}}\cdot \sqrt{n}$, where *n* refers to the number of averages or repetitions. However, we chose to compare CNR_eff_ because it reflects differences in the contrast mechanism of interest—STE-DWI vs. LTE-DWI—rather than specific protocols settings such as a number of averages used to acquire this particular set of data.

### Statistical Analysis

The differences in SIR and CNR_eff_ between LTE- and STE-DWI were compared using a paired Wilcoxon signed-rank test with a significance threshold of *p* = 0.05. Non-parametric tests were used because neither LTE nor STE values were normally distributed (one-sample Kolmogorov-Smirnov test independently on LTE and STE distributions at significance level *p* = 0.05).

## Results

### Patients

Twenty-five patients were analyzed. In this cohort, the most prevalent type was WHO IV glioblastoma (68%), followed by WHO III anaplastic astrocytoma (12%), and WHO II oligodendroglioma (12%). Their age was between 26 and 77 years with a mean ± SD of 61 ± 11 years. The overview of the demographics of the patients is summarized in Table 1. Individual patients are referred to as P1, P2, P3, etc. For visual comparison, we also show the images in two additional patients: in a patient without glioma-related MRI abnormalities (C1) and a patient with a non-enhancing WHO IV glioblastoma without DWI-hyperintensity (C2).

**TABLE 1:** Demographics table of the patients’ population characteristics.

		Frequency	Percentage
Number of patients		25	
Age	Median ± standard deviation	61 ± 11 years	
	Range (min – max)	26 – 77 years	
Sex	Male	21	84 %
	Female	4	16 %
Type	Oligodendroglioma (WHO II)	2	8 %
	Astrocytoma (WHO II)	1	4 %
	Diffuse astrocytoma (WHO II)	1	4 %
	Glioblastoma (WHO IV)	17	68 %
	Anaplastic astrocytoma (WHO III)	3	12 %
	Anaplastic Oligodendroglioma (WHO III)	1	4 %
Grade	WHO I	0	0 %
	WHO II	4	16 %
	WHO III	4	16 %
	WHO IV	17	68 %
IDH	Wild-type	16	64 %
	Mutated	5	20 %
	Unknown	4	16 %
MGMT	Methylated	8	32 %
	Non-methylated	8	32 %
	Unknown	9	36 %

*All patients are classified according to WHO 2016 classification (Louis et al., 2016).*

### Qualitative Analysis

**FIGURE 1:**
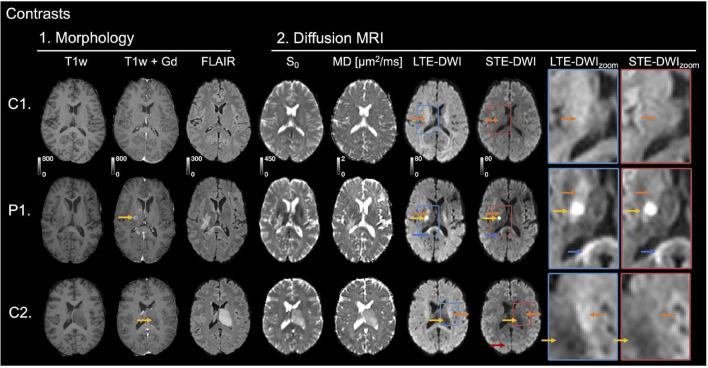
Investigated image contrasts. Three contrasts were related to morphology: T1w, T1w contrast-enhanced by Gadolinium (T1w+Gd), and T2w FLAIR. Four were related to DWI: *S*_0_, mean diffusivity (MD) and high *b*-value diffusion-weighted imaging (DWI) (*b* **=** 2,000 s/mm^2^) obtained with linear tensor encoding (LTE) and spherical tensor encoding (STE). Insets to the right show zoomed-in versions of LTE-DWI (blue frame) and STE-DWI (red frame). To simplify the comparison between STE-DWI and LTE-DWI, the signal intensities of the zoom-ins were averaged to the same average intensity value, but zoom-outs had the same signal intensity windows (this applies also to Figures 4, 5). The top row shows images from a male (C1) without MRI abnormalities except for vascular malformation (telangiectasia) in the right temporal lobe outside of evaluated regions. The second row shows a patient (P1, male, 52 years), who had an astrocytoma of grade II [mutated; isocitrate dehydrogenase (IDH)] in a similar slice as C1. The MRI was performed to confirm tumor progression. On the post-Gd contrast-enhanced T1-w image, an enhancing lesion can be seen (marked with yellow arrow). Both LTE-DWI and STE-DWI show a tumor-related hyperintensity (yellow arrows), but only STE-DWI attenuates the white matter surrounding the tumor (orange arrows). Apart from the obvious lesions, there is another hyperintensity with similar appearance on both LTE-DWI and STE-DWI (blue arrows). The third row shows the second patient example (C2, male, 57 years), who had a WHO IV glioblastoma (IDH wild-type). The tumor is non-enhancing on contrast-enhanced T1w+Gd image (yellow arrow) and does not show any tumor-related hyperintensity on neither LTE-DWI nor STE-DWI. However, the white matter tracts shifted by the adjacent tumor are attenuated with STE-DWI, but not LTE-DWI (orange arrows). Red arrow points to a fat artifact that is also visible at the same location in other maps.

The study investigated T1w images before and after administration of gadolinium, T2 FLAIR, *S*_0_, MD, LTE-DWI, and STE-DWI (Figure 1). Visually, the conspicuity of the tumor lesions was enhanced using STE-DWI compared to LTE-DWI (Figure 1, patient P1). That is because STE at *b* = 2,000 s/mm^2^ attenuates the white matter signal more compared to LTE. The attenuation of white matter can also be seen in a similar slice in a patient without relevant MRI abnormalities (Figure 1, C1), as well as in white matter tracts that are shifted by the adjacent tumor in the vicinity of a non-enhancing WHO IV glioblastoma (Figure 1, patient C2).

**FIGURE 2:**
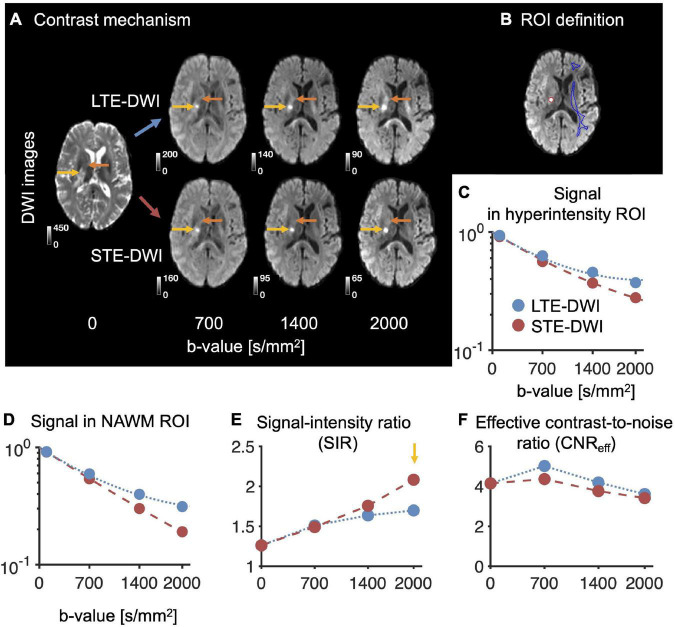
Illustration of STE-DWI and LTE-DWI contrasts across different *b*-values. Panel **(A)** shows that the contrasts with LTE-DWI and STE-DWI are similar at low *b*-values, but diverge with increasing diffusion encoding strength. Already at low *b*-values, a hyperintense lesion is visible with both LTE-DWI and STE-DWI (yellow arrows). At higher *b*-values, both the lesion and nearby white matter are hyperintensed with LTE-DWI (orange arrows), but not with STE-DWI, in which the white matter is attenuated. Panel **(B)** shows the definition of region-of-interests (ROIs). The red region delineates ROI of a hyperintensity, whereas the blue region a normally appearing contralateral white matter (NAWM). Panels **(C,D)** show signal attenuations in the hyperintensity and in NAWM, respectively. The dotted blue line corresponds to LTE-DWI, while dashed red line to STE-DWI. Panel **(E)** shows that the ratio of the signal intensity of the hyperintensity and the NAWM (i.e., the SIR-value) increases with the *b*-value and reaches higher values with STE-DWI than with LTE-DWI (yellow arrow). Panel **(F)** shows the contrast-to-noise ratio efficiency (CNR_eff_) in the hyperintensity and the NAWM. The CNR_eff_ decreases with increasing *b*-values, but it is non-zero even for *b*-value of 0 s/mm^2^ due to high T2w FLAIR signal in the tumor. This indicates that SIR may be a more intuitive quantity than CNR_eff_ for capturing these differences in contrast. Data are from patient P1 in Figure 1.

**FIGURE 3:**
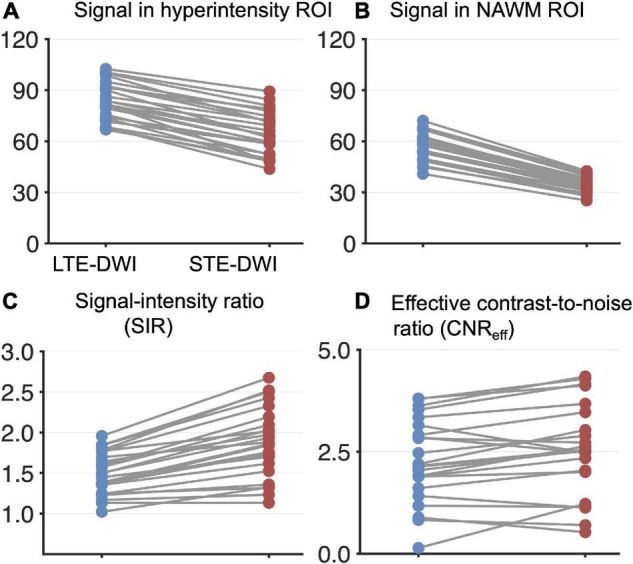
Quantitative analysis of LTE-DWI and STE-DWI at a *b*-value of 2,000 s/mm^2^ from 25 patients (each patient contributes with one pair of data points). Panel **(A)** shows signal differences of LTE-DWI (blue dots) and STE-DWI (red dots) in the hyperintensities. Panel **(B)** shows the corresponding results for the NAWM. Panel **(C)** shows the signal intensity ratio (SIR). Panel **(D)** shows CNR_eff_. Both the SIR and CNR_eff_ were significantly higher with STE-DWI than LTE-DWI (see main text for details).

**FIGURE 4:**
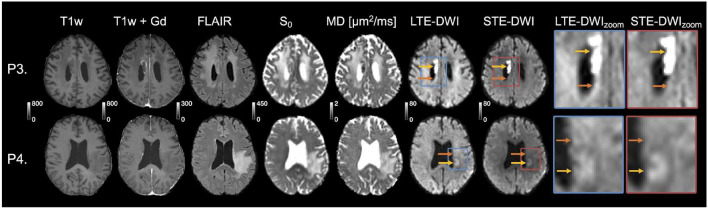
Tumor-related hyperintensities obscured by white matter. The top row (patient P3) shows images obtained to evaluate radionecrosis in a female (69 years old) diagnosed with anaplastic astrocytoma of grade III (wild-type IDH). The periventricular white matter (orange arrow) surrounding the tumor-related hyperintensity (yellow arrow) is visible with LTE-DWI, but is attenuated with STE-DWI. The hyperintensity is more clearly delineated with STE-DWI. The second row (P4) shows images obtained to confirm tumor progression in a female (71 years old) diagnosed with glioblastoma grade IV [methylated; O6-methylguanine-DNA methyltransferase (MGMT)]. A tumor-related hyperintensity (yellow arrow) can be seen with similar intensity as the surrounding white matter on LTE-DWI. With STE-DWI, the hyperintensity stands out more clearly (orange arrows) and could be missed out if only the LTE-DWI is inspected.

**FIGURE 5:**
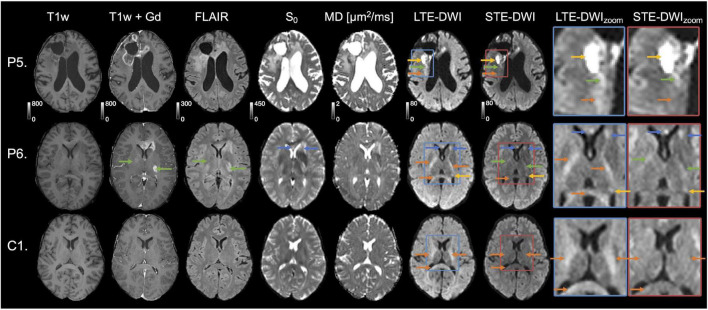
Interpretation challenges: Examples where inclusion of STE-DWI could improve diagnostic confidence. The top row (patient P5) shows images obtained after initiation of therapy (Optune) from a 62-year-old male diagnosed with glioblastoma grade IV (wild-type IDH, non-methylated MGMT). The tumor-related hyperintensity (yellow arrows) is with LTE-DWI surrounded by what appears to be hyperintense white matter (orange arrow). However, only parts of what may be interpreted as hyperintense white matter on LTE-DWI is attenuated with STE-DWI (orange arrow). The part closer to the main lesion remains hyperintensed with STE-DWI (green arrow), which suggests that it is a tumor-related hyperintensity. The middle row (P6) shows images obtained to confirm tumor progression in a 31-year-old male diagnosed with glioblastoma grade IV (mutated IDH). Several LTE-DWI and STE-DWI hyperintensities can be observed, however, STE-DWI suppresses those related to white matter (orange arrows) and, thereby amplifies the contrast of those that are not white-matter related. Out of these, two may be due to T2 shine-through effect (blue arrows). Some of the STE-DWI hyperintensities were “hidden” with LTE-DWI under the white matter-related hyperintensities (green arrows). The STE-DWI suggests that they may be tumor-related hyperintensities coming from tumor cells spreading along the white matter tracts. Note that these tracts are surrounded by Gd-enhancements and T2 FLAIR hyperintensity (green arrows). The bottom row shows images obtained in a subject (C1) without relevant MRI abnormalities in the region evaluated to illustrate that—unlike in the previous case—all white-matter-related hyperintensities seen with LTE-DWI disappear with STE-DWI (orange arrows).

The conspicuity of the hyperintensity increases with the *b*-value (Figure 2A, depicting patient P1 from Figure 1). Without diffusion encoding (*b* = 0 s/mm^2^), an elevated DWI signals predominately due to a visibly high T2 signal, although the signal is not considerably higher than the signal of the surrounding. As the *b*-value increases, the hyperintensity that is also due to low MD becomes visible. However, with increasing *b*-value in LTE-DWI, the white matter also becomes increasingly hyperintense relative to both cortical and deep gray matter. With increasing *b*-value in STE-DWI, the signal is attenuated faster in NAWM than in hyperintensity, which results in a stronger contrast (Figures 2B-D). The SIR of both LTE-DWI and STE-DWI increases with the *b*-value, but the increase is faster for STE than LTE at higher *b*-values (Figure 2E). Thus, STE-DWI at higher *b*-values more clearly isolates the tumor-related hyperintensity. The CNR_eff_ of both LTE-DWI and STE-DWI decreases with increasing *b*-value in this patient and is non-zero even in the image without diffusion encoding, although the elevated DWI signal is predominately due to high T2 signal (Figure 1, patient P1).

### Quantitative Analysis

We investigated the diffusion-weighted signal itself, as well as the SIR and CNR_eff_ across all the tumors. The average signal in STE-DWI and LTE-DWI at *b* = 2,000 s/mm^2^ is shown in Figure 3A for hyperintensities and in Figure 3B for NAWM. The signal difference between LTE-DWI and STE-DWI was higher in NAWM than in the hyperintensities, with a median difference (interquartile range) of 22.7 (19.6 – 24.9) in NAWM vs. 18 (15.4 – 22.5) in the hyperintensities. Thus, STE-DWI yields a relatively stronger attenuation of NAWM than of the hyperintensities. On average, it yielded a 26 % stronger attenuation (the ratio between the medians is 1.26).

The SIR, which is the ratio of signals between the hyperintensities and NAWM, was significantly higher for STE-DWI than LTE-DWI, with a median (interquartile range) of 1.9 (1.6 – 2.1) vs. 1.4 (1.3 – 1.7) (*p* < 10^–4^; paired Wilcoxon signed-rank test; Figure 3C). All of the cases displayed higher SIR on STE-DWI than on LTE-DWI, and STE-DWI increased SIR on average by 28%. In 10 of the 25 patients (40%) that had a hyperintensity unrelated to white matter, the SIR for STE-DWI was above 2, but with LTE-DWI, the SIR was below 2 for all patients.

The CNR_eff_ was significantly higher for STE-DWI than LTE-DWI, with a median (interquartile range) of 2.5 (2 – 3.5) vs. 2.3 (1.7 – 3.1) (*p* < 10^–3^; paired Wilcoxon signed-rank test; Figure 3D). The CNR_eff_ was higher on STE-DWI than on LTE-DWI in 70% of all cases. Note that Figure 2F shows a case that had a lower CNR_eff_ on the STE-DWI than the LTE-DWI, but despite this, we observe a clear increase in the conspicuity of the lesion.

### Improvements in Diagnostic Confidence

Figure 4 illustrates cases, where the radiological interpretation is clear from both LTE-DWI and STE-DWI. Both cases show a tumor-related hyperintensity embedded in white matter and illustrate how the contrast is improved with STE-DWI. In the top row (P3), the STE-DWI, and not LTE-DWI, results in suppressed periventricular white matter. The additional suppression obtained with STE-DWI increases the contrast between the lesion and the surrounding white matter. In the bottom row (P4), both STE-DWI and LTE-DWI show a tumor-related region with an elevated signal with similar intensity as the surrounding white matter, where the contrast between the tumor-related lesion and the white matter is slightly stronger with STE-DWI than with LTE-DWI.

Figure 5 illustrates two cases (P5 and P6), where STE-DWI provides a complementary contrast to LTE-DWI, as well as a patient without relevant MRI abnormalities (C1) as a reference. In the top row (P5), STE-DWI and LTE-DWI reveal different regions as hyperintense. One region is strongly hyperintensed in both LTE-DWI and STE-DWI (yellow arrow), while another region is weakly hyperintensed in LTE-DWI, but isointense in STE-DWI (orange arrow). A third region is weakly hyperintensed on both LTE- and STE-DWI (green arrows). This third region might be regarded as white-matter-related when viewing LTE-DWI only. However, the bright appearance of STE-DWI suggests that it is tumor-related. In the second patient (P6), LTE-DWI and STE-DWI show multiple regions as hyperintense, where some, but not all, may be considered as malignant tissue. Two regions are strongly hyperintensed on both LTE-DWI and STE-DWI (blue and yellow arrows). Three regions are hyperintensed on LTE-DWI but not on STE-DWI, as it suppresses white matter-related hyperintensities (orange arrows). Importantly, STE-DWI shows two hyperintensities (green arrows) that were not visible on LTE-DWI. This suggests that STE-DWI, unlike LTE-DWI, may visualize lesions that would otherwise be hidden in white matter. These two hyperintensities are also indicated in the T1w+Gd (gadolinium) and T2 FLAIR images (green arrows). The gadolinium enhancements also point to a malignant process and further support this interpretation. For comparison, the third row shows a slice from a patient without relevant MRI abnormalities (C1) in the same location, as that in the second patient. Again, we see white matter-related hyperintensities (orange arrows) in LTE-DWI that disappear and become isointense with the surrounding tissue in STE-DWI.

### Differences Between STE-DWI and LTE-DWI in MRI Without Abnormalities

**FIGURE 6:**
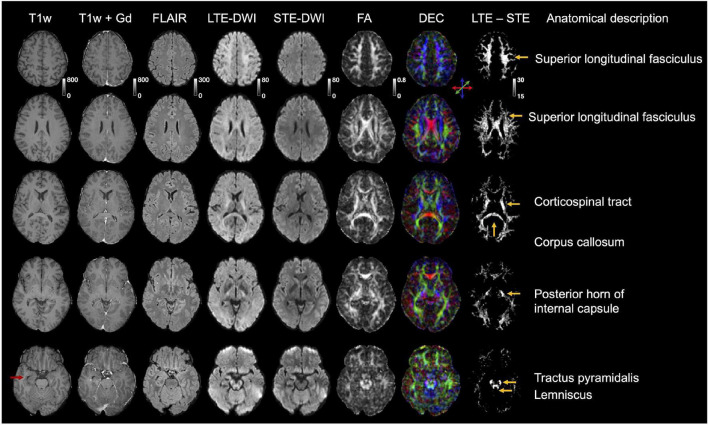
Illustration of the difference between LTE-DWI and STE-DWI in a patient without MRI brain abnormalities, except for a vascular malformation (telangiectasia) in right temporal lobe outside of evaluated regions (red arrow). This patient was previously referred to as C1 and is shown in several slices. Rightmost column shows difference maps between LTE-DWI and STE-DWI at *b*-value of 2,000 s/mm^2^ windowed such that only the highest differences are visible. The difference maps shows that the major white matter tracts are the most suppressed by STE-DWI (tract names shown in the righthand side of the figure). The LTE- and STE-DWI were shown with the same colormap (“windowing”). DEC = direction encoded color map.

To illustrate the differences between LTE-DWI and STE-DWI, we show their difference maps in a patient without relevant MRI abnormalities (C1) in Figure 6, along with indications of the description of anatomical locations. The differences are largest in major white matter tracts.

### Data Quality of STE-DWI

**FIGURE 7:**
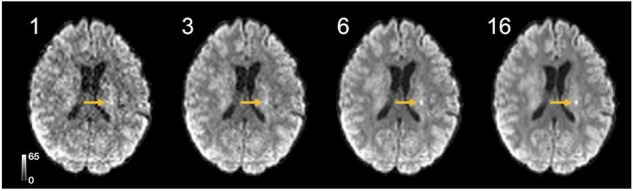
High-quality high *b*-value STE-DWI (*b* = 2,000 s/mm^2^) images can be obtained with short scan times and few averages. The tumor-related hyperintensity is visible with 3 averages (yellow arrows), but becomes clearer with 6 averages and yields an image quality similar to that obtained with 16 averages. With a repetition time of 5,000 ms, 6 averages can be acquired in 30 s.

We acquired STE-DWI with 16 averages, but Figure 7 shows that high-quality STE-DWI can be obtained with fewer averages. Comparing 1, 3, 6, and 16 averages, we see that the image quality markedly improves already with three averages, and that the difference in image quality between the images obtained with 6 and 16 averages is limited.

## Discussion

In this study, we compared the contrast mechanisms in glioma-related DWI hyperintensities obtained with STE-DWI and conventional LTE_DWI averaged across several directions at higher *b*-values (*b* = 2000 s/mm^2^). We found that STE-DWI improves the conspicuity of white matter-embedded tumor tissue compared with LTE-DWI. The finding was observed in multiple individual cases (Figure 1), and the potential diagnostic utility of STE-DWI was highlighted (Figures 4, 5). The improved conspicuity was also demonstrated quantitatively in Figure 3 using two quantities: the signal-intensity ratio—defined similarly to Seo et al. (2008)—and the contrast-to-noise ratio efficiency—applied similarly to Freitag et al. (2016). The former improved in all cases and in 70% of the cases. The improved conspicuity can be explained by opposite tendencies of glioma tumor tissue and white matter from the perspective of the signal attenuation (Figures 3A,B): STE-DWI attenuates anisotropic tissue components leaving only isotropic low-diffusion components contributing to the diffusion-weighed signal, which is prominent at higher *b*-values (Figure 2). Thus, STE-DWI emphasizes tissues, such as dense glioma tumors, with spherical and tightly packed cells that exhibit low diffusivity in all directions (Szczepankiewicz et al., 2015; Lundell et al., 2019; Tax et al., 2020; Vis et al., 2021). Signal attenuation in white matter was further illustrated in a patient without relevant MRI abnormalities (Figure 1, C1) and in a patient with a non-enhancing WHO IV glioblastoma without a DWI-hyperintensity (Figure 1, patient C2). Thus, STE-DWI attenuates healthy white matter to yield a contrast specific to hyperintensity.

STE-DWI may be relevant in the clinical settings for diagnosis or radiotherapy planning, where the presence of a pathology-related hyperintensity may improve detection or delineation. We expect the delineation and detection to be more accurate with STE-DWI because, unlike with LTE-DWI, a confounding factor (white matter) is suppressed as illustrated in Figure 5. The STE-DWI could even visualize information that is not accessible with conventional LTE-DWI. For example, tumor-related hyperintensities may exhibit similar signal intensity to white matter and a part of them is at risk of being missed or misinterpreted as a white matter if using only LTE-DWI (Figure 5, patient P5). Furthermore, STE-DWI could visualize tumor-related hyperintensities within white matter tracts (Figure 5, patient P6), which is a route of spreading for glioma tumor cells (Giese et al., 1996; Giovanna and Kaye, 2007). Including STE-DWI may reveal hyperintensities that, otherwise, could be missed (Figure 4, patient P4). Finally, the white matter that is shifted by adjacent tumor lesions is not depicted on STE-DWI, but only on LTE-DWI (Figure 1, patient C1), which could corroborate the differential diagnosis. The highest signal differences between LTE-DWI and STE-DWI were observed predominately in major white matter tracts (Figure 6). Hence, we expect STE-DWI to better show lesions “hidden” under white matter in these tracts and these anatomical locations could be of interest for applications of STE-DWI.

Conventional DWI at high *b*-values (3,000 s/mm^2^) has been shown to improve glioma conspicuity, compared with DWI at regular *b*-values (1,000 s/mm^2^) (Seo et al., 2008; Zeng et al., 2018). The analysis methods based on high *b*-values acquisitions, such as restricted spectrum imaging, may also increase glioma conspicuity (White et al., 2013). Another method of white matter suppression is based on subtraction the axial diffusivity (Freitag et al., 2016). It has shown to be useful in white matter tracts that have a coherent fiber direction but may not, unlike STE-DWI, suppress the signal adequately in fiber crossing regions. Tractography can also be applied to delineate glioma tumors from white matter (Nilsson et al., 2018a). However, the tractography based on LTE data only had limited accuracy (Maier-Hein et al., 2017) and might be improved by also including STE-data (Cottaar et al., 2020; Jeurissen and Szczepankiewicz, 2021). Other advanced techniques for improving DWI of gliomas include, for example, neurite orientation dispersion and density imaging (NODDI), which has been applied to differentiate glioma grade (Maximov et al., 2017; Li et al., 2019; Kadota et al., 2020) or to differentiate glioma from solitary brain metastasis together with mean apparent propagator MRI (MAP-MRI) (Mao et al., 2020). Microstructure models, such as VERDICT, have also been applied to map glioma microstructure (Zaccagna et al., 2019). Only a limited number of papers have based their investigation on data acquired with both LTE and STE (Nilsson et al., 2020; Li et al., 2021). What is different with the present approach is that it focuses on the contrast of STE-DWI itself, compared with LTE-DWI without modeling. It is, therefore, easy to integrate into a radiological workflow.

The STE-DWI has several advantages compared with LTE-DWI. First, STE-DWI produces images weighted by the isotropic diffusivity, whereas conventional LTE-DWI produces images weighted for the combination of isotropic diffusivity and diffusion anisotropy. This increases the specificity of the contrast. Note that STE-DWI is acquired using custom gradient waveforms for the encoding (Szczepankiewicz et al., 2021b) and, thus, cannot be obtained by post-processing of conventional LTE-DWI, such as by the trace of the diffusion tensor imaging (DTI). Second, STE-DWI is designed to be independent of the orientation of the object (rotationally invariant) that is already in the data acquisition step (Szczepankiewicz et al., 2021b), which also allows for greater flexibility in the design of imaging protocols (Nilsson et al., 2020). By contrast, in LTE-DWI, the signal must be acquired across sufficient directions to make it rotationally invariant (Mori and Van Zijl, 1995; Wong et al., 1995; Szczepankiewicz et al., 2019). Third, STE-DWI is feasible at common clinical platforms (Szczepankiewicz et al., 2019; Nilsson et al., 2020) and the scan times are generally short: we acquired a 6-average high *b*-value STE-DWI in just 30 seconds at an isotropic resolution of 2.3 mm^3^ (Figure 7). There are, however, also drawbacks. The major ones are that STE has higher demands on the gradient system and that it requires longer echo times (TE) and, thus, provides comparatively lower SNR. In particular, an encoding strength of *b* = 2,000 s/mm^2^ can be achieved in approximately 15 ms shorter echo time by using a stand-alone optimized conventional pulsed-gradient LTE-DWI sequence (Stejskal and Tanner, 1965), when compared to STE-DWI on a gradient system with maximal gradient amplitude of 80 m T/m and slew rate of 100 T/m/s (Szczepankiewicz et al., 2021b). In this study, we acquired a TE of 84 ms for both STE and LTE, which is close to the currently achievable minimal TE for the STE at *b* = 2,000 s/mm^2^ and this hardware configuration. This corresponds to an SNR reduction for STE-DWI of around 20%, under the assumption of exponential T2 signal decay in white matter with a T2 relaxation time of 80 ms (Whittall et al., 2002). The CNR_eff_ of LTE-DWI would, thus, increase by 20%. Lesions that are also hyperintense on T2w FLAIR images have longer T2-relaxation times and suffer less from this penalty. How the SIR would change for an optimized LTE-DWI sequence is harder to predict, as it depends on the relative difference in T2 between the hyperintensity and the white matter. Within age-associated white matter lesions, there is a substantial difference between the T2-relaxation time within and outside of axons, which leads to a considerable dependence of the diffusion-weighted signal on the TE (Lampinen et al., 2019). We expect this to also be the case for tumor-related hyperintensities in T2w images but are not aware of any detailed analysis. Note that diffusion-relaxometry techniques could be of interest here to add microstructure specificity (De Almeida Martins and Topgaard, 2018; Slator et al., 2021; Narvaez et al., 2022). This was, however, outside the aim of the present work, which was to study the contrast mechanism provided by STE-DWI without modeling or optimizing the imaging protocol.

The histological underpinnings of DWI- and T2/FLAIR-hyperintensities remain elusive, although it has been proposed that the pathophysiological mechanism of the reduced diffusivity in DWI-hyperintensities can be high the tumor cellularity (Chen et al., 2013; Laviolette et al., 2014). However, these may also appear to be due to prolonged T2 relaxation times in the tumor (T2-shine through effect). Even though the T2/FLAIR-hyperintensities, which are often in the tumor regions on MRI scans, coincide with DWI-hyperintensities, their pathophysiological mechanism at the tissue level in the tumor lesion could be different. It is proposed that these are associated with irreversible myelin or axonal loss, enlarged perivascular spaces due to lack of drainage of interstitial fluid, increased protein concentration that could lead to edema, or due to gliosis (Melhem et al., 1997; Ho et al., 2012; Haller et al., 2013).

We identified five limitations of the present study. First, the study lacks histological assessment of the tissue composition in the DWI-hyperintensities, and, thus, we cannot verify its histological correlate. Thus, the clinical usefulness of STE-DWI is only indicative at this point. Future studies, including comparisons with histological findings or targeted biopsies, would be highly beneficial. The second limitation is that we did not compare the conspicuity with other imaging modalities, such as perfusion MRI, or with metrics that can be extracted from the data by modeling, especially those related to tissue anisotropy. The third limitation is related to the acquisition protocols. The LTE-DWI was averaged over only 6 directions, which could lead to some residual rotational variance. However, the rotational variance is below 1% in structures with FA below 0.6 (Szczepankiewicz et al., 2019), and glioma tumors generally have low FA. For the STE-DWI, gradient waveforms were not compensated for concomitant gradient effects. These may introduce a position-dependent bias and future studies should use waveforms with minimized concomitant gradient effects (Szczepankiewicz et al., 2021b). Fourth, the contrast could also depend on the TE. The aim of the study was, however, to study the influence of the b-tensor shape on the image contrast. The fifth and last limitation is the limited sample size. Larger cohorts are needed to assess the improvements in tumor conspicuity in clinical settings.

## Conclusion

We present initial evidence that STE-DWI at high *b*-values enables a more specific assessment of tumor hyperintensities in glioma tumors by suppressing the confounding contribution from white matter.

## Data Availability Statement

The data analysis were processed by a software package for diffusion MRI available at: https://github.com/markus-nilsson/md-dmri (Nilsson et al., 2018b). Project-specific code, MRI protocol and waveforms are available at: https://github.com/jan-brabec/tensor_valued_gliomas_in_vivo.

## Ethics Statement

The project was approved by the local ethics committee-the Regional Ethical Review Board in Lund (2016/531, 2017/866 and 2018/993), and written informed consent was obtained from each volunteer according to recommendations of the declaration of Helsinki.

## Author Contributions

JB: conceptualization, investigation, formal analysis, writing - original draft, methodology, and writing - review and editing. FD: data curation, resources, and writing - review and editing. FS: conceptualization, data acquisition, investigation, software, methodology, and writing - review and editing. PB and BL: investigation writing - review and editing. AR: resources writing - review and editing. LK: funding acquisition writing - review and editing. C-FW: funding acquisition and writing - review and editing. PS: conceptualization, supervision, visualization, project administration, resources, methodology, funding acquisition, and writing - review and editing. MN: conceptualization, supervision, project administration, investigation, writing - original draft, writing - review and editing, and software, methodology. All authors have read and approved the final version of the manuscript.

## Conflict of Interest

MN declares ownership interests in Random Walk Imaging, and patent applications in Sweden (1250453-6 and 1250452-8), in the United States (61/642 594 and 61/642 589), and *via* the Patent Cooperation Treaty (SE2013/050492 and SE2013/050493). JB, MN, FS, and PS are inventors on pending patents pertaining to the methods presented herein in the United States (63/293 098). The remaining authors declare that the research was conducted in the absence of any commercial or financial relationships that could be construed as a potential conflict of interest.

## Publisher’s Note

All claims expressed in this article are solely those of the authors and do not necessarily represent those of their affiliated organizations, or those of the publisher, the editors and the reviewers. Any product that may be evaluated in this article, or claim that may be made by its manufacturer, is not guaranteed or endorsed by the publisher.

## Abbreviations

ADC: Apparent diffusion coefficient
AUC: Area under curve
CNR: Contrast-to-noise ratio
CNReff: Contrast-to-noise ratio efficiency
CSF: Cerebrospinal fluid
DEC: Direction encoded color map
dMRI: Diffusion magnetic resonance imaging
DTI: Diffusion tensor imaging
DWI: Diffusion-weighted image
FA: Fractional anisotropy
FOV: Field of view
FLAIR: Fluid-attenuated inversion recovery
IDH: isocitrate dehydrogenase
LTE: Linear tensor encoding
LTE-DWI: Diffusion-weighted image/imaging obtained with linear tensor encoding
MAP-MRI: mean apparent propagator MRI
MD: Mean diffusivity
MR: Magnetic resonance
MK: Mean kurtosis
MPRAGE: magnetization prepared rapid gradient echo
NODDI: neurite orientation dispersion and density imaging
ROI: Region-of-interest
SIR: Signal intensity ratio
STD: Standard deviation
STE: Spherical tensor encoding
STE-DWI: Diffusion-weighted image/imaging obtained with spherical tensor encoding
TE: Echo time
TR: Repetition time
WHO: World Health Organization.
